# Macrophage Efferocytosis as a Therapeutic Strategy in Intervertebral Disc Degeneration

**DOI:** 10.1111/cpr.70068

**Published:** 2025-06-09

**Authors:** Shijie Chen, Haijun Zhang, Zhaoheng Wang, Daxue Zhu, Yanhu Li, Yizhi Zhang, Dongxin Wang, Shuwei Chen, Huan Liu, Xuewen Kang

**Affiliations:** ^1^ Lanzhou University Second Hospital Lanzhou People's Republic of China; ^2^ Orthopaedics Key Laboratory of Gansu Province Lanzhou People's Republic of China; ^3^ The Second People's Hospital of Gansu Province Lanzhou People's Republic of China

**Keywords:** efferocytosis, intervertebral disc degeneration, macrophages, therapeutic strategy

## Abstract

In recent years, a growing number of studies have disclosed the substantial role of macrophages—key immune cells—in the pathological process of intervertebral disc degeneration. Researchers have categorised macrophage phenotypes into M1 and M2 polarisation, associating these polarisations with intervertebral disc degeneration. Essentially, macrophage phenotypes can be classified as either pro‐inflammatory or anti‐inflammatory. Induced by diverse factors, these distinct polarisation states exert contrary effects on disc injury and repair. Although numerous studies focus on the polarisation of macrophages and the cytokines they secrete in relation to intervertebral disc degeneration, these studies frequently neglect the relationship between the efferocytosis of macrophages and the progression of intervertebral disc degeneration. Efferocytosis is a specialised procedure in which phagocytes, such as macrophages, engulf and eliminate apoptotic cells. This process is crucial for maintaining tissue homeostasis and resolving inflammation. By effectively clearing these dying cells, efferocytosis helps prevent the release of potentially detrimental cellular contents, thereby facilitating healing and the resolution of inflammation. Simultaneously, macrophages digest the engulfed cell debris and release various cytokines that participate in tissue self‐repair. Therefore, this article presents an overview of the molecular mechanisms connecting macrophages and their efferocytosis activity to intervertebral disc degeneration, explores new directions for the utilisation of macrophages in the treatment of intervertebral disc degeneration, and discusses the future prospects for the development of therapeutic targets.

## Graphical abstract

Macrophages, integral to degenerated intervertebral discs, play a critical role in disc degeneration. By performing efferocytosis, they clear apoptotic cells. This article explores the process of efferocytosis, its molecular mechanisms, potential therapeutic implications for intervertebral disc degeneration and future strategies for targeted treatments.

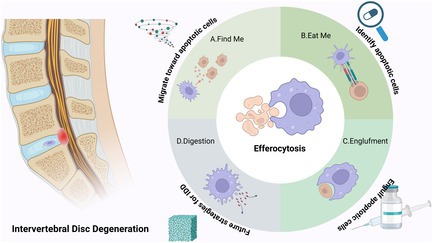

**Abbreviations**
β2GPI: beta‐2‐glycoprotein I
ACs: apoptotic cells
ADP: adenosine diphosphate
AF: annulus fibrosus
ATP: adenosine triphosphate
BAI1: brain‐specific angiogenesis inhibitor 1
BCG: Bacille Calmette‐Guérin
CEP: cartilage endplate
CEP: cartilaginous endplates
CHI3L1: chitinase‐3‐like protein 1
COX‐2: cyclooxygenase‐2
CX3CL1: fractalkine
DAMPs: damage‐associated molecular patterns
ECM: extracellular matrix
EIL: efferocytosis‐induced lactate
EIMP: efferocytosis‐induced macrophage proliferation
EMAP II: endothelial monocyte‐activating polypeptide II
ERK: extracellular signal‐regulated kinase
FAO: fatty acid oxidation
FKN: fractalkine
G2A: G protein‐coupled receptor G2A
Gas6: growth arrest‐specific 6
GM‐CSF: granulocyte‐macrophage colony‐stimulating factor
GPCR: G protein‐coupled receptor
HBD: heparin‐binding domain
IDD: intervertebral disc degeneration
IGF: insulin‐like growth factor
IL‐1ra: IL‐1 receptor antagonist
IL‐8: interleukin‐8
IL‐R: IL‐1 receptor
iNOS: inducible nitric oxide synthase
IVD: intervertebral disc
JNK: c‐Jun N‐terminal kinase
LAMPs: lifestyle‐associated molecular patterns
LBP: low back pain
LPC: lysophosphatidylcholine
LPS: lipopolysaccharide
LRP: LDL receptor‐related protein
M1CM: M1‐conditioned media
M1‐Exos: exosomes derived from M1 macrophages
M2CM: M2‐conditioned media
MCP‐1: monocyte chemotactic protein 1
MerTK: Mer tyrosine kinase
MFG‐E8: milk fat globule‐EGF factor 8
MMP‐3/9: Matrix Metalloproteinases‐3/9
MSCs: marrow mesenchymal stem cells
NP: nucleus pulposus
NPCs: nucleus pulposus cells
PAF: platelet‐activating factor
PANX1: plasma membrane channel Pannexin 1
PC: phosphatidylcholine
PCD: programmed cell death
PECAM‐1: platelet endothelial cell adhesion molecule‐1
PFKFB2: enzyme 6‐phosphofructo‐2‐kinase/fructose‐2,6‐bisphosphatase 2
PGE2: prostaglandin E2
PtdSer: phosphatidylserine
RA: rheumatoid arthritis
RAGE: receptor for advanced glycation end‐products
RBC: red blood cells
RP S19: ribosomal protein S19
S1P: sphingosine‐1‐phosphate
S1P1: G protein‐coupled receptor S1P receptor 1
S1PR1–5: five S1P receptors
SIRPα: SHPS‐1
SphK1: sphingosine kinase
SPMs: specialised pro‐resolving mediators
TGF‐β: transforming growth factor‐β
TIM: T cell immunoglobulin mucin receptors
TMEM16F: transmembrane protein 16F
TNFR1: tumour necrosis factor receptor‐1
TNFSF14: TNF superfamily member 14
TNF‐α: tumour necrosis factor‐α
TSP‐1: thrombospondin‐1
UDP: uridine diphosphate
UTP: uridine triphosphate
VEGF: vascular endothelial growth factor
WISP‐1: WNT1‐inducible signalling protein 1

## 1 Introduction

Low back pain (LBP), a serious condition, impacts the lives of many middle‐aged and elderly individuals, with a lifetime incidence of 70%–85% [1]. It also places a substantial burden on both healthcare systems and the economy [2]. The exact cause of LBP remains unclear, but intervertebral disc degeneration (IDD) is considered one of the primary contributors, affecting approximately 26%–42% [3]. IDD is a common spinal disease that leads to the deterioration of the structure and function of the intervertebral disc (IVD). Generally, IDD does not present with obvious symptoms [4]. However, as IDD progresses, it can eventually lead to disc herniation, sciatica, spondylolisthesis and even spinal stenosis, which are direct causes of chronic disability [5]. Additionally, as IDD continues to develop, it can present with neurological symptoms such as neuralgia, numbness, intermittent claudication, muscle weakness and even paralysis [5].

The primary pathological features of IDD are extracellular matrix (ECM) degradation, cell apoptosis and inflammation, which are interrelated and interdependent [6]. Among these, apoptosis is a significant response, characterised by the death of nucleus pulposus cells (NPCs) in the IVD, accumulation of dead cells and release of their contents. These contents, such as damage‐associated molecular patterns (DAMPs) and lifestyle‐associated molecular patterns (LAMPs), can affect the body, triggering inflammatory responses and leading to disease states [7, 8]. Gruber and Hanley's research revealed a significant increase in apoptosis rates within degenerated human IVDs [9]. Many IVD cells undergo programmed cell death (PCD) during the degeneration process.

Efferocytosis is an effective method for eliminating apoptotic cells (ACs) and plays a critical role in resolving inflammation. Nearly all types of cells have the ability to phagocytise ACs. Research has shown that IVD cells possess the capability to act as phagocytes, demonstrated by their ability to engulf latex beads and ACs [10]. However, some cells are specialised for devouring ACs and are known as professional phagocytes [11]. Macrophages are the most common professional phagocytes, capable of rapidly and continuously engulfing and processing multiple cell corpses [12, 13]. In recent years, growing evidence has highlighted the crucial role of macrophages, as key immune cells, in the pathogenesis of IDD. Researchers have identified a connection between macrophage polarisation into M1 or M2 phenotypes and IDD. The mechanisms by which macrophages exert unipolar or dual regulatory effects in IDD are being further explored. However, most studies have focused on macrophage polarisation or the relationship between macrophage cytokines and disc degeneration, often overlooking the role of macrophage efferocytosis in the progression of IDD. Therefore, we provide an overview of the molecular mechanisms of macrophages and efferocytosis in disease, and their association with IDD. Additionally, we explore new directions for macrophage‐based therapies and discuss the future prospects for developing therapeutic targets for IDD.

## 2 Macrophage Efferocytosis

### 2.1 What Is Efferocytosis?

**FIGURE 1:**
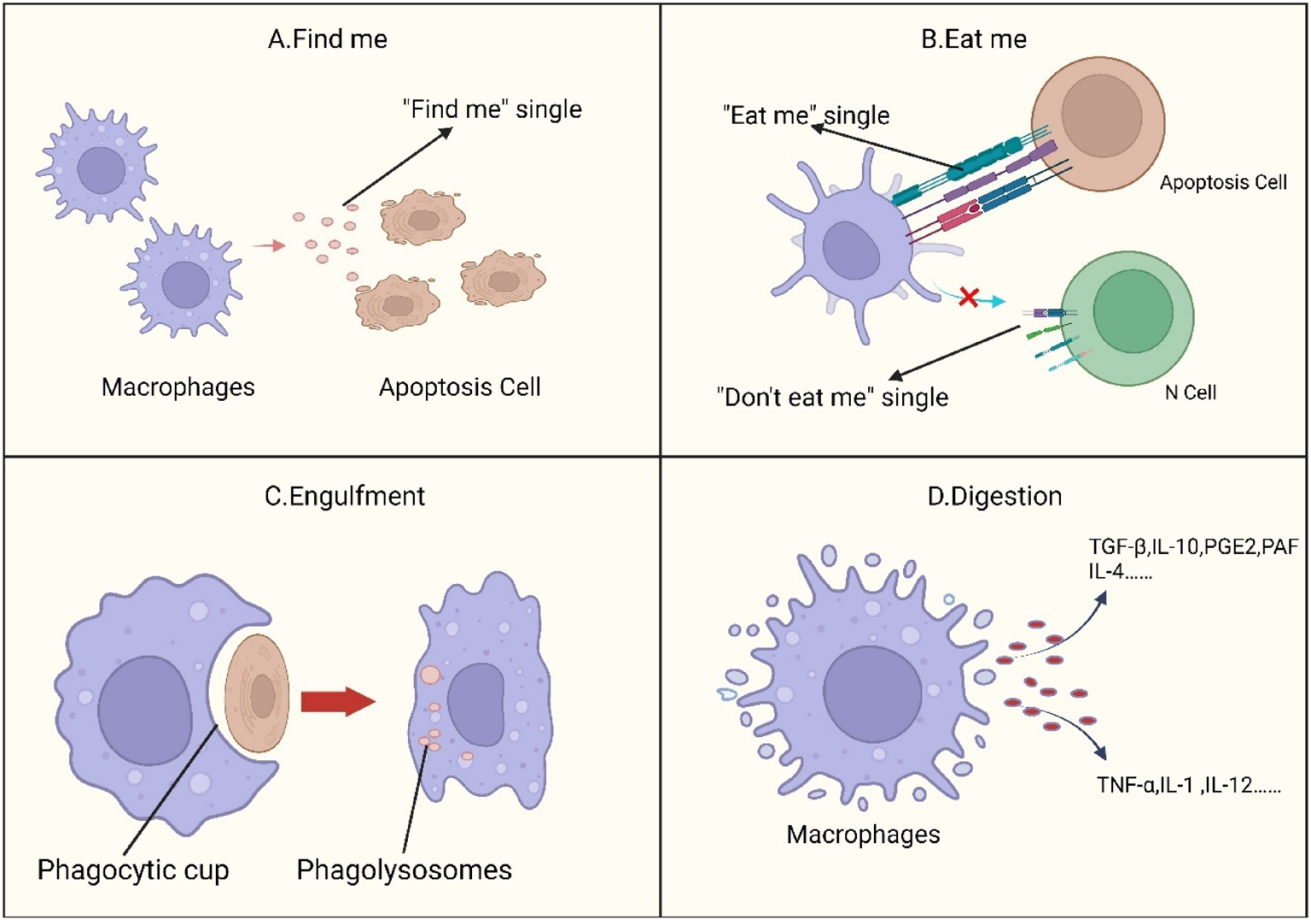
Efferocytosis. (A) Apoptosis involves the decomposition of cells into apoptotic bodies to prevent inflammation, during which ACs emit ‘find me’ signals, such as membrane lipids, nucleotides and CX3CL1, to recruit and activate macrophages for efficient clearance. (B) Macrophages recognise and phagocytose dying cells by distinguishing ACs, which display specific ‘eat me’ signals, from normal cells, which exhibit ‘don't eat me’ signals to prevent their engulfment. (C) Macrophages recognise ‘eat me’ signals, initiating the phagocytic phase and triggering a series of signalling events involving Rho‐GTPases and cytoskeletal remodelling, which leads to the engulfment of ACs. (D) During the digestion phase, macrophages break down engulfed ACs, resulting in increased release of certain cytokines that trigger a series of changes, while the release of other cytokines decreases, initiating various activities. Image created with BioRender.com with permission.

Cells in the human body are constantly renewing, meaning that cells are continually dying—about 1 million per second, typically through PCD or apoptosis [14]. In numerous physiological and pathological processes, macrophages are recognised as the principal phagocytes responsible for the majority of cell clearance [15]. Consequently, macrophages have been the focus of extensive research. Macrophages clear unwanted cells, including those that have undergone PCD or apoptosis [16]. This clearance process is called efferocytosis. Derived from the Latin ‘efferre’, meaning ‘to carry to the grave’, this biological term refers to the process in which phagocytic cells, such as macrophages or dendritic cells, engulf and eliminate ACs [11]. Macrophages identify and ingest ACs to prevent inflammation or other harmful reactions. This process is vital for immune system balance and helps eliminate unnecessary or damaged cells, supporting tissue repair and regeneration [17]. Issues or failures in phagocytosis can result in the buildup of ACs, which may in turn trigger chronic inflammation and autoimmune responses [18, 19, 20, 21, 22]. Efferocytosis is a complex process that involves many signal pathways and interactions between cells, ensuring the effective and safe removal of ACs. Extensive research on efferocytosis not only deepens our understanding of how the immune system regulates itself and repairs tissues but also opens up possibilities for discovering new treatment approaches. This research is highly valuable in clinical settings, offering promising avenues for treating inflammatory conditions and supporting tissue wellbeing. As shown in Figure 1, efferocytosis involves the interaction between ACs and macrophages. We summarise this process into four parts: ‘Find me’, ‘Eat me’, engulfment and digestion.

### 2.2 Macrophages Are Recruited by ACs—‘Find Me’ Singles

Apoptosis is a programmed cell death process in which specialised enzymes and molecules decompose the cell into apoptotic bodies, thereby preventing an inflammatory response [23, 24]. However, even in tissues with high cell turnover rates, such as bone marrow or the thymus, ACs are rarely observed. This indicates that ACs emit signals at the earliest stages of apoptosis to alert and attract macrophages. When ACs are not adjacent to macrophages, phagocytes must actively seek them out. During this process, ACs release various ‘find me’ signals to recruit and activate nearby macrophages, thereby promoting the efficient clearance of apoptotic bodies [25, 26]. These signals encompass membrane lipids [27, 28], nucleotides [29] and the chemokine fractalkine (CX3CL1) [30], with their importance varying according to the type and condition of the cell.

#### 2.2.1 Lipid Signals Released by AC Function as ‘Find Me’ Signals

Reports indicate that the membrane lipids lysophosphatidylcholine (LPC) and sphingosine‐1‐phosphate (S1P) serve as specific signals of apoptosis [31, 32]. These lipids are synthesised and released by specific enzymes, and they can affect the survival and function of macrophages. LPC is recognised as the earliest identified lipid ‘find me’ signal [27]. Lauber et al. demonstrated that LPC can inhibit the migration of macrophages to the supernatant of apoptotic MCF7casp3 cells in a dose‐dependent manner [27]. However, it remains unclear whether LPC is generated intracellularly, extracellularly or both during apoptosis, as disturbed membrane structures are susceptible to the activity of secreted phospholipase A2 [33]. Although G2A is recognised as the receptor target for LPC, the role of G2A in phagocyte recognition of LPC has been a matter of debate. Naoka Murakami et al. suggested that LPC acts as an antagonist rather than an agonist in regulating the proton‐dependent activation of G2A [34]. Moreover, Michael R. Elliott et al. found that macrophages did not migrate to purified LPC within a certain concentration range, challenging the concept of LPC as a ‘find me’ signal and indicating the need for further research to elucidate LPC–G2A signalling recognition.

Another lipid ‘find me’ signal, S1P, is produced from sphingosine by sphingosine kinase (SphK1) and regulates efferocytosis through its interaction with the G protein‐coupled receptor (GPCR) S1P receptor 1 (S1P1) [35]. S1P is primarily synthesised within cells and subsequently crosses the plasma membrane to perform its critical biological functions extracellularly [36]. Gude et al. demonstrated that S1P is released immediately following cell apoptosis, potentially generated and released via the upregulation of SphK1 [28]. As a ‘find me’ signal during efferocytosis, S1P induces macrophages to engulf ACs, thereby preventing their necrosis. Although five S1P receptors(S1PR1–5) are known, the specific GPCR involved in the recruitment of phagocytes to ACs remains unclear.

#### 2.2.2 Nucleotides Released by AC Act as ‘Find Me’ Signals Recruiting Phagocytes

Extracellular nucleotides released by AC also function as ‘find me’ signals [29]. These nucleotides play vital roles in intercellular communication and signalling pathways, being released into the surrounding environment in response to cell damage, stress, bacterial infection or other harmful stimuli. Nucleotides such as adenosine triphosphate (ATP), adenosine diphosphate (ADP), uridine triphosphate (UTP) and uridine diphosphate (UDP) are implicated in this process. Elliott et al. observed that nucleotides are released following various types of apoptotic stimuli, and confirmed that among the four extracellular nucleotides studied, ATP and UTP are identified as the key ‘find me’ signals released by ACs [29].

Chekeni et al. found that inhibiting the plasma membrane channel Pannexin 1 (PANX1) reduces nucleotide release from ACs and decreases recruitment of monocytes [37]. Conversely, overexpression of PANX1 enhances nucleotide release from ACs and boosts recruitment of phagocytes. Wherefore, PANX1 is recognised as a pivotal mediator in regulating the release of nucleotides or ‘find‐me’ signals from ACs [37]. Elliott et al. found that ATP and UTP regulate efferocytosis by binding to P2Y2 receptors on monocytes and macrophages [29]. Marques‐da‐Silva et al. discovered that the tight binding of extracellular nucleotides to P2 receptors (P2X1 or P2X3) enhances macrophage binding to apoptotic bodies, thereby increasing macrophage engulfment of ACs [38]. Koizumi et al. demonstrated that UDP activation can initiate P2Y6 receptor signalling, triggering microglial phagocytosis [39]. Overall, extracellular ATP and UTP are recognised as DAMPs. They act not only as ‘find me’ signals for efferocytosis through activation of corresponding P2Y receptors but also enhance phagocyte receptor expression. This promotes the recognition and efficient removal of these signals by phagocytosis [40].

#### 2.2.3 Chemokines Serve as ‘Find Me’ Signals in Efferocytosis

Chemokines are a class of chemokines (approximately 8–17 kDa) that mediate inflammation [41]. Based on the number and arrangement of conserved cysteine residues in their amino‐terminal peptide sequences, chemotactic factors are divided into four main subfamilies: C, CC, CXC and CX3C [42]. However, among the many human chemokines, only the transmembrane chemokine CX3CL1—the sole known member of the CX3C chemokine family—is widely recognised as a ‘find‐me’ signal that mediates macrophage exocytosis [43]. Belonging to the CX3C family, CX3CL1, also referred to as fractalkine (FKN) or neurotactin, actively promotes macrophage chemotaxis towards ACs by interacting with its receptor CX3CR1 [44, 45]. CX3CL1 is a 90 kDa membrane‐associated chemotactic factor released in particles exposed to phosphatidylserine (PtdSer) during cellular apoptosis [33]. As a chemotactic factor and cell adhesion molecule, CX3CL1 is swiftly released from apoptotic lymphocytes through caspase and Bcl‐2 regulatory pathways to attract macrophages [30]. Moreover, CX3CL1 enhances the clearance of ACs [46]. While some researchers propose that CX3CL1 may be the sole chemokine involved in efferocytosis, it is likely that among the four families of chemokines, CX3CL1 alone does not exclusively mediate the attraction of macrophages to ACs. The CX3C chemokine differs from the CXC chemokine at three conserved N‐terminal cysteine‐proximal amino acid residues. Despite differing by only one amino acid, certain CXC chemokines also exhibit chemotactic activity. CXCL12, alone or in synergy with multiple chemokine family members, enhances immune cell migration [47]. Chemokines such as CCL2 and CCL5, which share roles with CX3CL1 in immune regulation, cell migration and inflammation, may also contribute to efferocytosis [48, 49]. In addition to CX3CL1, other chemokines involved in efferocytosis require further investigation.

Chemotactic factors can also regulate phagocyte cytoskeleton, enhance phagocyte receptor expression and activate digestion mechanisms [50]. In conclusion, chemokines play a crucial role as ‘find‐me’ signals in the context of apoptotic cell clearance, facilitating efficient macrophage migration and contributing to immune system homeostasis. However, unlike the well‐defined pathways involving ATP nucleotide release through pannexin channels during apoptosis‐induced macrophage attraction, specific mechanisms of CXCL1 release from ACs remain to be elucidated.

#### 2.2.4 Proteins and Other Possible Molecules Act as ‘Find Me’ Signals to Recruit Macrophages

Previously, it was mentioned that LPC was discovered a long time ago. Even earlier, researchers found that dimers of ribosomal protein S19 (RP S19) released from ACs could induce monocyte phagocytosis [51]. During apoptosis, RP S19 dimerises and binds to the C5a receptor, recruiting macrophages as its chemoattractant ligand in a paracrine manner, facilitating their phagocytosis of ACs [51, 52].

Some proteins are also released during apoptosis and induce monocyte phagocytes to aggregate towards ACs, such as endothelial monocyte activating polypeptide II (EMAP II) [53]. The receptor for EMAP II has not been explicitly identified in the available search results. However, it is recognised that EMAP II can augment the expression of tumour necrosis factor receptor‐1 (TNFR1) in cancer cells [54]. This elevation in TNFR1 enhances the cells' responsiveness to tumour necrosis factor‐α (TNF‐α), suggesting that TNFR1 could serve as a receptor or an intermediary in mediating the effects of EMAP II in specific scenarios. However, whether this relationship exists and plays a role between macrophages and ACs needs further verification. Another potential protein factor is thrombospondin‐1 (TSP‐1) and its cleaved 26 kDa heparin‐binding domain (HBD) [55]. TSP‐1 and HBD are produced and released during apoptosis and have been shown to be chemotactic for monocytes [56]. TSP receptors (aVβ3, CD36) interact with TSP‐1 to mediate macrophage recognition of apoptotic neutrophils [57]. However, the specific evidence for TSP‐1 as a ‘Find me’ signal in efferocytosis is not sufficient, and the exact chemotactic mechanism remains unclear.

In addition to the aforementioned protein molecules, there is another intriguing yet not fully understood apoptotic derivative: apoptotic microvesicles. These spherical vesicles, approximately 0.18 μm in size, also exhibit chemotactic activity towards monocytes, which disappears upon their removal [58]. Similar to ectosomes, apoptotic vesicles involved in efferocytosis require further research to elucidate the specific phagocyte sensors and mechanisms. Additionally, studies have shown that Fas/CD95‐induced production of monocyte chemotactic protein 1 (MCP‐1) and interleukin‐8 (IL‐8) enhances the chemotactic response of phagocytes to ACs [59]. Researchers have also noted that intracellular pathogens can generate complement‐dependent ‘Find me’ signals using virulence factors. However, the specific applications of this mechanism require further investigation [60]. As shown in Table 1, we have summarised the information related to ‘find me’ signals.

**TABLE 1:** ‘Find me’ signal.

Classification	‘Find me’ signal	Receptors for signalling and bridging molecules	References
Lipid signalling	LPC	G protein‐coupled Receptor GA2	[27, 31, 32, 33, 34, 61]
	S1P	S1P 1	[28, 35, 36, 62]
Nucleotide	ATP and UTP	P2 receptors	[29, 37, 38, 39, 40, 63, 64, 65]
Chemokines	CX3CL1	CX3CR1	[41, 42, 43, 44, 45, 46, 50, 66]
Protein molecules	S19	C5a receptor	[51, 52, 67]
	EMAP II	TNFR1	[53, 54]
	TSP‐1and HBD	Integrin aVβ3, CD36	[55, 56, 57]
Other possible signals	Apoptotic vesicles		[58]
	MCP‐1 and IL‐8		[59]
	Intracellular pathogens		[60]

### 2.3 The Recognition Stage of Efferocytosis—‘Eat Me’ and ‘Don't Eat Me’

Triggered by ‘find me’ signals, macrophages migrate towards ACs, initiating the ‘engulfment phase’. This phase involves the recognition and phagocytosis of dying cells by macrophages. During the recognition phase, macrophages discriminate ACs from normal cells by identifying specific signals displayed by ACs. ACs expose signals known as ‘eat me’ signals, while normal cells exhibit ‘Don't eat me’ signals that prevent interaction with phagocytes, thereby safeguarding them from engulfment.

#### 2.3.1 Non‐Protein Molecules in the ‘Eat Me’ Signal

The most widely recognised and discussed ‘eat me’ signal is the translocation of PtdSer from the inner leaflet to the outer leaflet of the plasma membrane [16, 68]. In live cells, PS is not evenly distributed. It is predominantly localised to the inner leaflet of the cell membrane and within membrane structures formed during endocytosis, a localisation governed by a series of enzymes that work in concert [69]. During the process of apoptosis, the asymmetrical distribution of phospholipids across the plasma membrane is lost, resulting in the translocation of PS, typically confined to the inner leaflet, to the outer leaflet, thereby exposing it to the extracellular environment [69]. However, not all externalised PS molecules have the same biological roles. Specifically, it is the PS externalised through the Xkr8 pathway that acts as an ‘eat‐me’ signal, facilitating the process of efferocytosis [70]. PS is directly recognised by specific receptors on the phagocyte membrane, including brain‐specific angiogenesis inhibitor 1 (BAI1), T cell immunoglobulin mucin receptors (TIM)‐1 and TIM‐4, stabilin‐2 and the receptor for advanced glycation end‐products (RAGE), among others [71, 72, 73, 74]. To date, more than 15 cell surface proteins have been identified that mediate the binding of macrophages to apoptotic cell PS, either directly or indirectly via a bridging molecule [75]. In addition to the lipid‐based ‘eat‐me’ signal PS, which is widely acknowledged and investigated, the glycocalyx—a sugar coat on the surface of human cells—also has a role in ‘eat‐me’ signalling. Sialic acids, also referred to as Sias, which are terminal sugars on the cell surface glycans, show a decrease in their connection with terminal Sias and galactose on the apoptotic blebs during the early stages of apoptosis. Furthermore, the exposure of fucose residues on the derived blebs promotes phagocytosis by phagocytes [76]. Thus, the compositional changes in the glycocalyx on the surface of ACs appear to serve as an ‘eat‐me’ signal on the surface of apoptotic lymphocytes.

#### 2.3.2 Protein Molecules in ‘Eat Me’ Signal

In the realm of protein molecules, membrane‐anchored proteins play a pivotal role as ‘eat me’ signals during efferocytosis. Calreticulin facilitates the clearance of ACs by binding to and activating the LDL receptor‐related protein (LRP) on phagocytes [77]. Protein S enhances the ability of macrophages to engulf ACs by binding to PS expressed on the surface of these cells [78]. Growth arrest‐specific 6 (Gas6), a ligand for MerTK with sequence homology to Protein S, also binds to PtdSer and interacts with the phagocytic receptor MerTK [79]. Milk fat globule‐EGF factor 8 (MFG‐E8), a secretory glycoprotein produced by macrophages, specifically binds to ACs by recognising PtdSer, thus serving as an ‘eat me’ molecule [80]. Beta‐2‐glycoprotein I (β2GPI) and membrane cofactor protein I, both acting as specific bridging molecules for PS, are regarded as ‘eat‐me’ signals [81, 82].

#### 2.3.3 ‘Don't Eat Me’

The text mentions that PS can be externalised in non‐apoptotic situations without attracting macrophage phagocytosis, involving ‘don't eat me’ signals that prevent macrophages from engulfing normal cells. Healthy cells can evade efferocytosis through ‘don't eat me’ signals, also known as ‘save me’ signals [26]. The discovery of the interaction between CD47 (integrin‐associated protein) on cells and SIRPα (SHPS‐1) has revealed the tolerance of tissue macrophages towards cells that possess CD47 [83]. In ACs, alterations or loss of CD47 can reinitiate the clearance of cells by macrophages [77]. Besides CD47, CD31, also known as platelet endothelial cell adhesion molecule‐1 (PECAM‐1), transmits a ‘don't eat me’ signal to prevent phagocytic cells from engulfing closely neighbouring living cells [84]. In humans, CD300a exhibits specificity in recognising PS, thereby inhibiting efferocytosis [85]. However, the CD300 family comprises multiple members, and the impact of each on efferocytosis requires individual analysis [86]. Siglecs, or sialic acid‐binding immunoglobulin‐type lectins, bind to specific sialoglycan complexes, potentially regulating macrophage efferocytosis, as seen with CD33/Siglec‐3, CD22/Siglec‐2 and Siglec‐10 [87]. Le et al. demonstrated that the removal of glycocalyx by the disassembly of the cortical cytoskeleton from apoptotic bodies exposes the essential ‘eat me’ signals required for efferocytosis [88]. Therefore, the thick layer of glycocalyx formed by glycoproteins on the cell membrane, by reducing the proximity of ligands to receptors on the phagocytic cell membrane, can also be considered a ‘don't eat me’ signal [89].

### 2.4 Feeding of Macrophage—Phagocytosis

After undergoing the phases of chemotaxis and recognition, macrophages converge upon and scrutinise ACs, culminating in the act of phagocytosis, akin to a gourmet moment for the cell. This stage of engulfment stands in contrast to endocytosis, where vesicles cloaked in clathrin capture smaller particulates, and pinocytosis, the cellular uptake of soluble elements. Phagocytosis distinguishes itself by the ingestion of comparatively larger particles, those exceeding a diameter of 0.5 μm, effectively ‘devouring’ them into the cellular fold [90]. In the elimination phase of ACs, macrophages predominantly engage in phagocytosis, setting aside other cellular uptake mechanisms. The interaction between the ‘eat me’ signals and phagocytic receptors on macrophages sets off a cascade of signalling events, thereby activating the phagocytic response [79]. Given that PS serves as the primary ‘eat me’ signal, this review mainly delineates PS as the key initiator of phagocytosis. The externalisation of PS and its subsequent interaction with PS receptors on macrophages catalyse the process of engulfment.

Vorselen has meticulously detailed the activation of PS receptors such as Ba0i1 with Rac1, the downstream molecule Gulp of the PS receptor Megf10 activating Rho GTPase, and integrins activating Rho GTPases. These are among several direct and indirect receptors of PS that initiate a signalling pathway centred on Rho‐GTPases. This pathway, which includes Rac1, Rac2, RhoA and RhoG, triggers a cascade of cytoskeletal remodelling signals, leading to the reorganisation of the phagocytic cell's cytoskeleton [75]. Upon receptor activation, actin undergoes extensive polymerisation, which then drives the formation of pseudopodia, enveloping the apoptotic cell to form a structure rich in actin, known as the phagocytic cup [91]. Intracellular signalling in phagocytes relies on evolutionarily conserved pathways, such as CED‐1/CED‐6/CED‐7 and CED‐2/CED‐5/CED‐12, which activate Rho family GTPases like Rac1 to orchestrate cytoskeletal rearrangement and apoptotic cell engulfment, while additional receptors and mediators refine the process to maintain immune balance and suppress inflammation [92]. Phagosomes possess membrane‐binding proteins that can recruit and fuse with lysosomes, forming mature phagolysosomes, leading to the degradation of the phagosome contents [93, 94]. The fusion of phagosomes with lysosomes results in the formation of phagosome–lysosome complexes. During this process, enzymes from the lysosomes are released into the phagosomes, initiating the degradation of the contents within. Once the degradation is complete, the phagosome‐lysosome complex enters a dissipation phase, gradually restoring the homeostasis within the macrophage and permitting further phagocytosis [50].

## 3 The Function of the Substances Post‐Digestion in Efferocytosis

Efferocytosis, often referred to as the ‘silent death’ of ACs, involves macrophages engulfing and digesting these cells. This process results in the release of cytokines, which serve to alleviate inflammation, suppress autoimmune reactions, and induce tissue repair responses.

### 3.1 Efferocytosis: Suppressing Inflammation and Facilitating Tissue Repair

Efferocytosis is not merely a critical biological process for identifying and eliminating dead cells within the body; it is also a vital physiological mechanism that activates a series of anti‐inflammatory and tissue repair signalling pathways. Through this process, the generation of anti‐inflammatory cytokines is promoted, providing the potential for repair and regeneration of damaged tissues. Consequently, this maintains the body's equilibrium and a state of health. When macrophages phagocytise ACs and process them, there is an increase in the secretion of anti‐inflammatory and immune‐regulating cytokines such as IL‐10. Simultaneously, there is a decrease in the secretion of pro‐inflammatory cytokines, including TNF‐α, IL‐1 and IL‐12, contributing to the suppression of inflammation and promotion of tissue repair [95, 96, 97, 98]. The clearance of ACs triggers the secretion of IL‐10, which in turn activates the STAT3–IL10–IL6 autocrine–paracrine loop. This enables macrophages to maintain their clearance capabilities, which likewise aids in the resolution of inflammation [99]. IL‐10 modulates cytokines to alleviate inflammation, restrict antigen presentation and T‐cell activity, while also inhibiting the induction of nitric oxide synthase and the production of nitric oxide, thereby maintaining immune homeostasis [100]. IL‐10 not only promotes repair through its anti‐inflammatory properties but also directly participates in the cellular repair process. It exhibits reparative activity by regulating the ECM and fibroblast functions, thereby contributing to tissue restoration [101, 102]. IL‐10 also promotes tissue homeostasis by facilitating cellular differentiation, notably by upregulating BMP‐2 and BMP‐6 to drive mesenchymal cell differentiation into chondrocytes [103].

Transforming Growth Factor‐β (TGF‐β) and IL‐10 are considered primary mechanisms of anti‐inflammatory phagocytosis, with their interaction exerting a suppressive effect on inflammation. TGF‐β1, Prostaglandin E2 (PGE2), and Platelet‐Activating Factor (PAF) production increase during efferocytosis, subsequently inhibiting the production of pro‐inflammatory cytokines through autocrine/paracrine mechanisms [104]. Macrophages accelerate the resolution of lipopolysaccharide (LPS)‐induced pulmonary inflammation by recognising PS on the surface of ACs and relying on TGF‐β [105]. Furthermore, TGF‐β signalling has been found to promote the efferocytic function of IL‐10‐dependent macrophages [106]. The expression of Granulocyte–Macrophage Colony‐Stimulating Factor (GM‐CSF), Leukotriene C4 and Thromboxane B2 is also suppressed in human monocyte‐derived macrophages during efferocytosis. Macrophages that have engulfed ACs release anti‐inflammatory active lipids [107]. Additionally, efferocytosis can directly induce the resolution of inflammation through specialised pro‐resolving mediators (SPMs) [108].

During the various stages of autophagy, a series of distinct yet overlapping genes are activated, including multiple genes from the SLC family. These genes not only drive the glycolysis process but also the lactate release promoted by the SLC16A1 gene further contributes to the formation of an anti‐inflammatory tissue environment [109]. Zhang et al. found an interplay between fatty acid oxidation (FAO), mitochondrial respiration, and inflammation during the metabolic decomposition of ACs in autophagy [110]. The act of efferocytosis—engulfing and removing dying cells—triggers an anti‐inflammatory response in macrophages. This is achieved by augmenting the levels of long‐chain fatty acids within the macrophages, which in turn activate the respiratory chain and lead to the generation of metabolic signalling intermediates, such as NAD+, as per reference [110]. Moreover, the study elucidates how macrophages orchestrate anti‐inflammatory reprogramming and contribute to organ repair by modulating cellular metabolism and electron transport.

### 3.2 Efferocytosis Influences Macrophage Activation and Polarisation

Efferocytosis not only triggers the release of anti‐inflammatory agents but also exerts a significant impact on the overall modulation of immune responses through the mediation of macrophage polarisation. It has been demonstrated that efferocytosis leads to an upregulation in the secretion of IL‐10, TGF‐β and IL‐4, cytokines that are known to induce a shift in macrophages towards an M2 phenotype. In contrast, the release of factors associated with M1 polarisation, such as IL‐1β, TNF‐α and IL‐12, is inhibited during efferocytosis. The augmented levels of IL‐10 and TGF‐β act as catalysts for M2C macrophage polarisation, while the decreased levels of IL‐1β and TNF‐α are indicative of a shift away from M1 polarisation. This intricate balance of cytokine expression indicates that efferocytosis can suppress M1 macrophage polarisation, thereby favouring a transition towards an M2 phenotype. In osteosarcoma, MerTK‐mediated efferocytosis has been shown to steer macrophages towards M2 polarisation through the p38/STAT3 signalling pathway [111]. In contrast to their role in inflammation, M2‐like macrophages within tumours act as facilitators of cancer progression, aiding in tumour growth, invasion, and metastasis. Research indicates that thymosin alpha‐1 (Tα‐1) is capable of reversing the M2 polarisation of macrophages induced by efferocytosis through the activation of the TLR7/SHIP1 pathway [112]. The combination of Tα‐1 and Thalidomide can significantly reduce the levels of IL‐10 and enhance the function of CD4+/CD8+ T cells, effectively inhibiting the growth of breast cancer.

### 3.3 Efferocytosis Induces the Proliferation of Macrophages

During efferocytosis, macrophages release metabolites from phagosomes that degrade ACs, such as nucleic acids. These metabolites activate catabolic pathways, sustaining the macrophages' phagocytic activity. Gerlach et al. indicate that nucleotides derived from AC can stimulate macrophage proliferation pathways [113]. This phenomenon is termed Efferocytosis‐Induced Macrophage Proliferation (EIMP). The authors found that nucleic acids derived from ACs are hydrolysed by phagolysosomes during efferocytosis. The nucleotides produced from this hydrolysis initiate the AC‐nucleotide‐mTORC2 signalling pathway, which promotes the proliferation of macrophages that facilitate dissolution.

Efferocytosis also stimulates a temporary increase in glycolysis within macrophages, a process contingent upon the swift activation of the enzyme 6‐phosphofructo‐2‐kinase/fructose‐2,6‐bisphosphatase 2 (PFKFB2). The activated glycolysis, mediated by lactate, upregulates MerTK and LRP1, fostering on‐going efferocytic activity [114]. Concurrently, EIMP stimulates the production of efferocytosis‐induced lactate (EIL), with the lactate‐driven metabolic pathway stimulating macrophage proliferation [115].

## 4 Macrophages: Multifaceted Sentinels in IVD Health and Diseases

Macrophages, the body's versatile sentinels, are omnipresent across a myriad of tissues, endowed with multifunctional capabilities. These include the engulfment and elimination of pathogens, combating viral and bacterial threats, thwarting tumour proliferation and orchestrating immune responses alongside tissue reconstruction. In the realm of IDD, macrophages assume a dual role: they are instrumental in the removal of compromised tissue and, under certain conditions, can instigate inflammatory reactions that intricately influence disc integrity. The study of the interaction between IVDs and macrophages contributes to a deeper understanding of the pathogenesis of disc degeneration.

### 4.1 Macrophages in IDD: The Increasing Immune Cells With Degeneration

The IVD is the fibrocartilaginous part of the ‘three‐joint complex’, composed of the adjacent vertebral bodies' annulus fibrosus (AF), NP and cartilaginous endplates (CEP) [116]. The NP is enveloped by the lateral AF and capped by the superior and inferior EPs. This structure protects the NP from immune tolerance during foetal development, rendering the IVD an avascular organ with immune privilege, akin to other immune‐privileged organs such as the eyes and brain [117]. In a healthy NP, immune cells or inflammatory cytokines have not been detected [118]. During the pathological process of IDD, both passive and active immune privilege barriers are compromised through various mechanisms [119]. When the immune barrier between the IVD and the immune system ruptures, macrophages are recruited to the exposed, degenerated NP [120]. Immune exposure typically occurs when the disc's AF or longitudinal ligaments are damaged, as well as when the CEP fractures. At this juncture, the physical and molecular immune barriers are crucial for maintaining the immune privilege status of the NP. If these barriers are compromised, not only may macrophage activity intensify, but it may also lead to the growth of blood vessels and nerves into the NP, disrupting the immune barrier and triggering an immune response, resulting in enhanced chemotaxis and infiltration of macrophages [121]. In rat NP tissue grafts, the presence of macrophages within the transplanted NP tissue indicates that the NP has a role in recruiting macrophages [122]. Additionally, it has been demonstrated that increased vascularisation may facilitate the entry of immune cells into the IVD [123, 124].

Numerous studies have demonstrated the presence of macrophages in IVD. Macrophages are key regulators in the initiation, progression, and resolution of inflammation, a role they also play in IVD [125]. Research has shown that inflammatory cells expressing the phagocytic marker CD68 are abundant in degenerated and herniated IVD tissues, indicating that phagocytes, or macrophages, are indeed present in IVD and play a significant role [118, 126]. Healthy human IVDs are considered to be immune‐privileged, with no CD68‐marked cells observed in the IVD of foetuses, infants and adolescents [118]. In degenerated IVD, the presence of macrophage markers does not increase with the severity of disc degeneration. However, there is a notable increase in macrophages in areas of the IVD that are structurally irregular and defective. This suggests that the quantity of macrophages is related to the extent of disc degeneration [127]. In a study of 205 patients with IVD protrusion, 25% were found to have a significant presence of macrophages without the detection of other inflammatory cells [128]. Furthermore, the prevalence of CC chemokine receptor (CCR) 7+ macrophages (M1) and Cd163+ (M2) macrophages increases with age and degeneration [127]. A correlation between macrophages and IDD has also been observed in mouse injury models. In a mouse model of lumbar IVD degeneration induced by puncture injury, infiltration of F4/80‐ir macrophages was noted on the fourth day and persisted within the IVD for up to 12 months post‐injury [129]. Macrophages have been confirmed to exist in degenerated IVD. In human degenerated NP tissue, higher expressions of M1 and M2 macrophage polarisation markers CCR7 and CD206 are observed [130].

**FIGURE 2:**
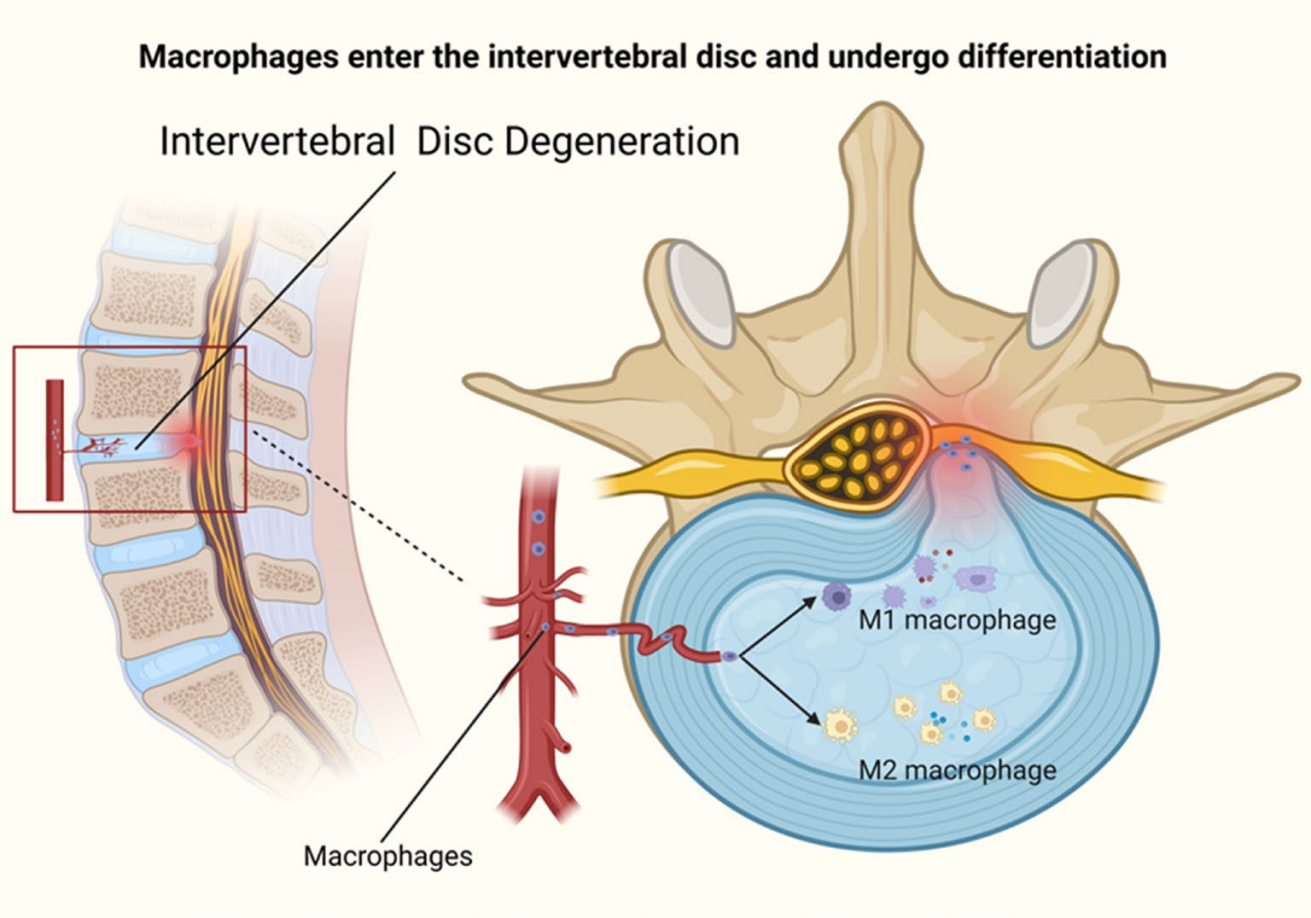
Macrophages in the intervertebral disc. The immune and physical barriers of the degenerated IVD are compromised, leading to the chemotactic migration of macrophages into the degenerated disc. These macrophages are then influenced by various cytokines to differentiate into M1 and M2 macrophages.

Macrophages in degenerated IVD include not only recruited macrophages but also resident macrophages that have been found within the IVD [131]. Recent studies indicate the presence of resident macrophages in both mouse and human IVD [127, 131, 132]. Yokozeki et al. identified a heterogeneous population of CD206− and CD206+ macrophages in mouse IVD [133]. Despite this finding, the scientific community still lacks comprehensive insights into the presence, ontogeny, and functional roles of resident macrophages within IVD. As shown in Figure 2, in degenerated IVD, compromised immune and physical barriers allow macrophages to migrate into the disc, where cytokines influence their differentiation into M1 and M2 macrophages.

### 4.2 Macrophage Polarisation in IDD: Mechanisms and Therapeutic Potentials

Macrophages are dynamic immune cells capable of altering their phenotype and functions in response to diverse stimuli, a process termed macrophage polarisation. Research delineates that macrophages can undergo polarisation into two distinct phenotypes: the classically activated M1 macrophages, which are pro‐inflammatory, and the alternatively activated M2 macrophages, which exhibit anti‐inflammatory properties. In 2000, Mills defined M1 and M2 based on the observation that macrophages from Th2 strains were more readily activated by IFN‐γ or LPS to produce NO compared to those from Th1 strains, in Balb/c and C57BL/6 mice [134]. Although some researchers have proposed a tri‐colour model to classify macrophages into three categories—classically activated, wound‐healing, and regulatory macrophages [135], there is also a viewpoint that discourages the use of the term ‘regulatory’ macrophages [136]. Despite criticisms that the M1/M2 paradigm may oversimplify the complexity of macrophage functions, the M0–M1–M2 classification system continues to be instrumental in elucidating the multifaceted roles of macrophages, offering a simplified yet informative framework for grasping the essentials of macrophage polarisation.

#### 4.2.1 The Impact of M1 Macrophages on IDD

M1 macrophages, also known as classically activated macrophages, were named by GB Mackaness in the 1960s to describe the pathogen antigen‐dependent but non‐specific enhanced bactericidal activity of macrophages upon secondary exposure to Bacille Calmette‐Guérin (BCG) and Listeria [137]. The hallmark of M1 macrophages is the expression of TLR2, TLR4, MHC‐II, CD80/CD86 and the production of pro‐inflammatory cytokines such as TNF‐α, IL‐1β, IL‐6, IL‐12 and IL‐23, as well as reactive oxygen and nitrogen species like NO and ROS [138, 139]. The polarisation of macrophages to the M1 phenotype can be best identified by the surface expression of CD64 and CD80 [140].

Li et al. discovered that the polarisation of M1 macrophages can induce the occurrence of IDD [130]. In vitro studies demonstrate that when NPCs are co‐cultured with M1‐conditioned media (M1CM), the M1 macrophages not only suppress NPCs proliferation but also enhance the secretion of pro‐inflammatory cytokines (TNF‐α, IL‐1β, IL‐6 and IL‐12), chemokines, and matrix metalloproteinases [130]. Dou et al. found that melatonin mitigates the polarisation of M1 macrophages through the SIRT1/Notch signalling pathway, thus reducing inflammation‐induced damage to NPCs [141]. Furthermore, M1 macrophages are implicated in more than just the initiation of inflammatory responses; exosomes derived from M1 macrophages (M1‐Exos) have been identified as accelerants of the ageing process in NP cells via the LCN2/NF‐κB signalling pathway [142]. Simultaneously, exosomes released by degenerating NPCs exhibit an antagonistic effect, promoting the polarisation of macrophages towards the M1 phenotype [143].

Recent studies on M1 macrophages have shed light on their pivotal role in the progression of IDD. Researchers have observed a marked increase in the infiltration of M1 macrophages and the secretion of pro‐inflammatory cytokines within the degenerating discs. These insights have spurred the development of targeted therapies aimed at modulating the M1 macrophage phenotype and its associated inflammatory activity to potentially halt or reverse IDD. One promising approach involves the use of a composite hydrogel scaffold imbued with dual‐drug/slow‐release PLGA microspheres that carry IL‐4 (IL‐4‐PLGA) and kartogenin (KGN‐PLGA). This innovative scaffold has demonstrated the ability to prompt an early transition of macrophages from the M1 phenotype, thereby exerting a therapeutic effect on IDD [144]. Despite these advancements, the interaction between M1 cells and NP cells, particularly the cellular pathways involved, is still not fully elucidated in scientific research. Delving into this field is crucial for uncovering the intricate cellular interactions and inflammatory responses involved in IDD.

#### 4.2.2 Phenotypes of M2 Macrophage and IDD

M2 macrophages, known as alternatively activated or wound‐healing macrophages, are key in fostering Th2 responses and are beneficial in dampening inflammation and promoting tissue repair and healing [145]. The presence of CD206+ macrophages (M2 macrophage) has been observed in degenerative NP in humans and mice [146]. Cytokines of M2 macrophages protect the disc by restraining cell proliferation and anabolic metabolism while encouraging the release of anti‐inflammatory agents [130]. Additionally, macrophages play a crucial role in disc degeneration and repair. M2‐conditioned media (M2CM) protects against IDD by stimulating cell proliferation, ECM synthesis, and inhibiting inflammation, apoptosis, and the senescence of NPCs [147].

M2‐polarised macrophages represent a diverse population, categorised into subgroups—M2a, M2b and M2c—based on their response to various inducers [148, 149]. Recent studies have expanded this classification to include the M2d subtype [150, 151, 152]. M2a macrophages, which are induced by interleukins IL‐4 or IL‐13, are often termed ‘wound‐healing’ macrophages due to their ability to express markers like Arg1, Ym1 and Fizz1 [153, 154]. M2a macrophages are characterised by elevated expression of the mannose receptor (MR, also known as CD206), the IL‐1 receptor (IL‐R) and chemokine CCL17. These cells facilitate tissue repair by secreting a suite of pro‐fibrotic mediators, including TGF‐β, Insulin‐like Growth Factor (IGF) and fibronectin, as indicated in references [150, 155]. Intriguingly, co‐culturing NPCs with M2a macrophages in vitro reveals that M2a cells potentiate catabolic processes. They do so by secreting chitinase‐3‐like protein 1 (CHI3L1), which activates the extracellular signal‐regulated kinase (ERK) and c‐Jun N‐terminal kinase (JNK) signalling pathways. This leads to an upregulation of matrix‐degrading enzymes, namely Matrix Metalloproteinases‐3 and 9 (MMP‐3 and MMP‐9), while concurrently downregulating the expression of matrix synthesis genes, such as aggrecan and type II collagen [156].

M2b macrophages, termed regulatory macrophages, are elicited in response to immune complexes coupled with TLR agonists such as LPS or IL‐1 receptor co‐agonists, including IL‐1β and IL‐6. This interaction markedly upregulates the expression of chemokine CCL1 and the TNF superfamily member 14 (TNFSF14) [148, 157, 158]. M2b macrophages are identified by their specific markers, CCL1 and LIGHT, and a spectrum of non‐specific markers such as IL‐10, CCL2, CD163, CD64, CD86, SPHK1, TNF‐α, and IL‐6 [157]. M2b macrophages are instrumental in Th2‐driven immune regulation and the modulation of inflammation [159]. Notably, the literature lacks focused research on the correlation between M2b macrophages and IDD.

M2c macrophages(alternatively activated or deactivation macrophages) are stimulated by interleukins such as IL‐10, TGF‐β, or glucocorticoids. These cells are prolific producers of cytokines IL‐10 and TGF‐β, and chemokines such as CCL16, CCL18, and CXCL13 [160, 161]. M2c macrophages are distinguished by high levels of cell surface markers CD163, Mer tyrosine kinase (MerTK), and Tie2 [162]. Compared to M2a, co‐culture of M2c macrophages with NP cells enhances NPC proliferation and migration, and promotes the synthesis and secretion of matrix proteins in NPCs [163].

Macrophage polarisation is not merely a dichotomy of M1 and M2 states but a dynamic spectrum reflecting their intricate and adaptable phenotypic and functional diversity. This complexity demands a more detailed comprehension in both investigative and clinical frameworks [164]. In the waning stages of acute inflammation, a distinct population of resolution‐phase macrophages has been isolated. These cells express mannose receptors and produce anti‐inflammatory cytokines like IL‐10 and arginase 1, yet they retain some prototypical M1 attributes, including the expression of cyclooxygenase‐2 (COX‐2) and inducible nitric oxide synthase (iNOS) [165]. In a parallel observation, macrophages from mice infected with *Streptococcus pyogenes* mount a classical M1 response without iNOS expression, while transcripts linked to M2 macrophage anti‐inflammatory actions, such as IL‐1 receptor antagonist (IL‐1ra) and IL‐10, are elevated. This highlights the need for further investigation into macrophage classifications within humans. A balanced interplay between M1 and M2 macrophages has been shown to facilitate the healing and regeneration of IDD, a finding corroborated by analogous research in osteoarthritis [166]. The equilibrium between M1 and M2 polarisation emerges as an auspicious avenue for prospective IDD studies. Delving into this equilibrium could uncover novel biomarkers and therapeutic targets, propelling the innovation of treatments tailored to IDD. The specific markers and cytokines associated with macrophages are summarised and integrated in Table 2.

**TABLE 2:** Macrophage polarisation and IDD.

Macrophage phenotypes	Specific markers	Cytokines	Effects on IDD	References
M1	CD80, CD86, CD16/32, MHCII, iNOS	TNF‐α, IL‐1β, IL‐6, IL‐12/23, NO, ROS	Inhibits NPC proliferation and promotes inflammation	[137, 138, 139, 140, 141, 142, 143, 144]
M2a	CD206, MHCII, IL‐1R, Dectin‐1	TGF‐β, IGF, Fibronectin	Promotes matrix catabolism and inhibits matrix synthesis in NP cells	[153, 154, 155, 156]
M2b	CD206, MHCII, CD86	CCL1, TNFSF14	No study	[148, 157, 158, 159]
M2c	CD206, CD163	IL‐10, TGF‐β, CCL16/18, CXCL13	Enhances NPCs proliferation and migration, and promotes the synthesis and secretion of matrix proteins	[160, 161, 162, 163]

## 5 Potential Therapeutic Strategies Targeting Macrophage Efferocytosis in IDD

**FIGURE 3:**
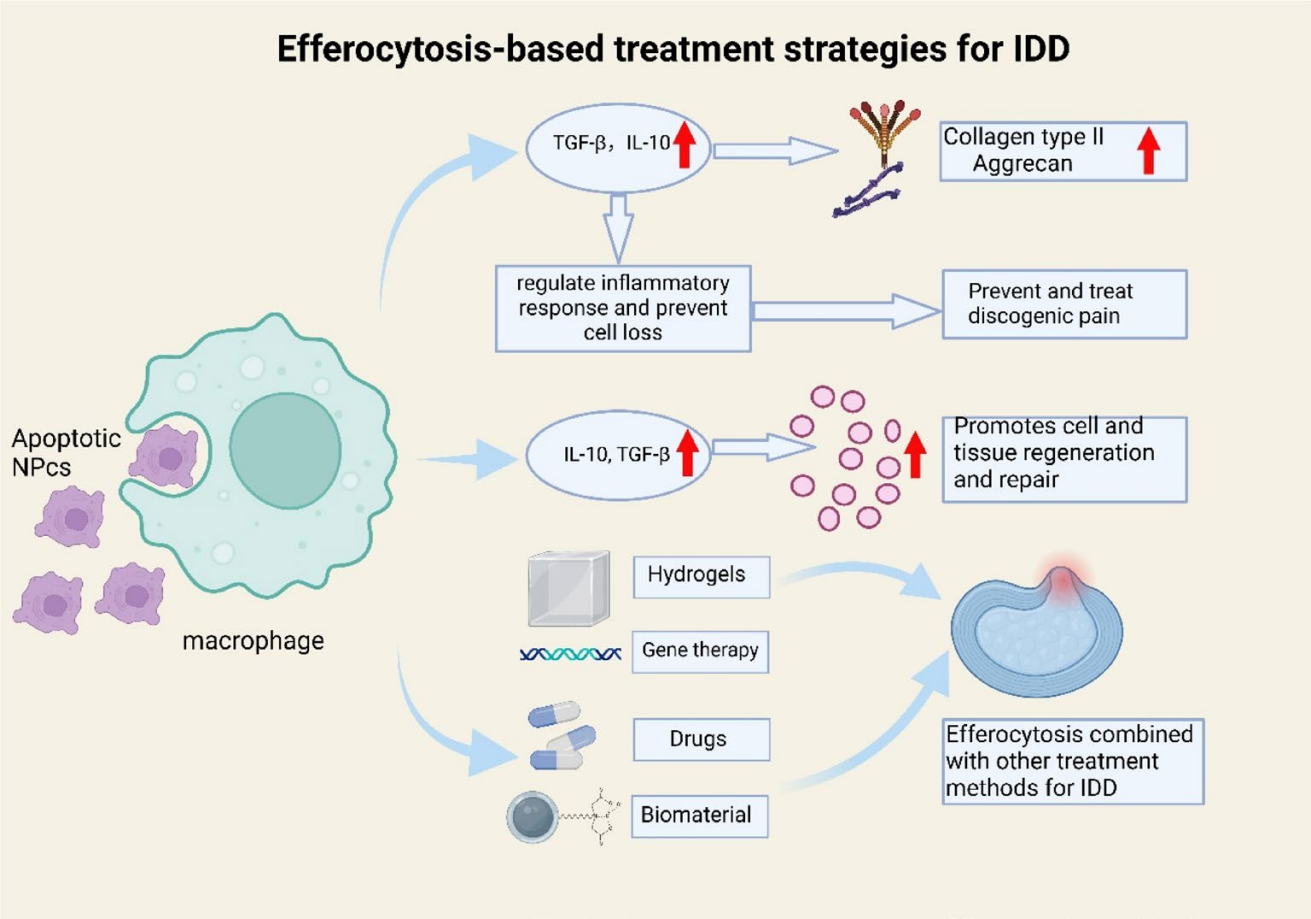
Possible therapeutic directions of efferocytosis for IDD. This figure outlines the possible therapeutic role of macrophage efferocytosis in IDD, reducing inflammation, promoting tissue repair and enhancing disc regeneration. Hypoxia‐driven autophagy further supports efferocytosis, while biomaterials, gene therapy and drugs (e.g., dexmedetomidine, statins) offer potential treatment strategies.

Efferocytosis, a sophisticated biological phenomenon involving the precise recognition and effective clearance of ACs by macrophages, plays a crucial role not only in maintaining tissue structural stability and functional equilibrium but also in the self‐repair process following tissue damage. The regulation of efferocytosis is especially crucial in the complex pathology of IDD. Through in‐depth research and precise modulation of efferocytosis, we hope to develop new therapeutic strategies that could not only slow down the degenerative changes in the IVD but also potentially offer new solutions for treating associated chronic pain and functional impairments. The potential therapeutic approaches for IDD that involve macrophage efferocytosis, along with the associated treatment methods, are summarised in Figure 3.

### 5.1 Efferocytosis: Alleviating Inflammation, Reducing Cytokine Release and Mitigating Pain

Efferocytosis leads to an increased secretion of IL‐10 and TGF‐β. It has been confirmed that exogenous IL‐10 treatment can suppress inflammatory responses in IVD, enhance the protein levels of type II collagen and aggrecan, and inhibit the activation of p38 MAPK, thereby offering therapeutic benefits for disc degeneration [167]. Inhibition of p38 MAPK activation typically results in the suppression of cellular oxidative stress and apoptosis [168]. Concurrently, TGF‐β effectively protects and repairs IVD tissue by promoting matrix synthesis, inhibiting degradation, regulating inflammatory responses and preventing cell loss [169]. Previous discussions have also highlighted the anti‐inflammatory effects of efferocytosis, indicating that macrophages, by engulfing ACs, not only reduce the number of these cells but also suppress the inflammatory response and the release of inflammatory cytokines.

The onset of discogenic pain is closely associated with the exacerbation of inflammation. The inflammatory response not only stimulates the accelerated degeneration of the IVD but may also induce the growth of nociceptive nerve fibres into the disc area, a process known as neoinnervation [170]. This abnormal nerve growth could lead to aberrant conduction of pain signals, thereby triggering or exacerbating the sensation of pain. Therefore, enhancing efferocytosis to regulate the inflammatory response may represent an effective therapeutic strategy for IDD.

### 5.2 Promoting Cell and Tissue Regeneration and Remodelling Through Efferocytosis

Enhancing the ability of macrophages to clear ACs, efferocytosis not only alleviates the inflammatory burden but also addresses IDD, which primarily includes the gradual reduction of nutrient supply to the IVD and excessive destruction of the ECM, a chronic process. Recent studies have revealed the significant role of IL‐10 in regulating ECM and fibroblast functions, surpassing its traditional anti‐inflammatory activity. IL‐10 can promote axonal regeneration in the nervous system, involving the modulation of STAT3 and NF‐κB signalling pathways [171]. Macrophage‐derived IL‐10, by activating CREB signalling, fosters the secretion of WNT1‐inducible signalling protein 1 (WISP‐1), which in turn drives the proliferation of epithelial cells [172]. In stem cell therapy for IDD, the administration of allogeneic bone marrow mesenchymal stem cells (MSCs) has been found to upregulate IL‐10 and downregulate TNF‐α, suggesting a potential interaction between IL‐10 and disc cell regeneration [173]. Efferocytosis serves not only to clear dead cells but also to activate cytokines and growth factors, promoting repair activities of cells such as fibroblasts and endothelial cells. In the heart, it triggers the production of vascular endothelial growth factors (VEGF) A and VEGFC, contributing to myocardial repair [174, 175]. Efferocytosis may also enhance the synthesis of the disc matrix. Another study found that monocyte‐derived macrophages, under the mediation of TGF‐β, phagocytising apoptotic debris, stimulated primary fibroblasts to produce collagen [176]. From this analysis, we can conclude that bolstering autophagy may accelerate the tissue repair process, aiding in the restoration of the structural integrity and functional efficacy of the IVD. Moreover, it could create a more favourable microenvironment for the natural recovery and regeneration of the disc.

### 5.3 Potential Enhancement of Efferocytosis in IVD Under Hypoxic Conditions

The NP is the central part of the IVD, surrounded by the AF arranged in a ring. The blood supply from the subchondral bone can reach the cartilage endplate (CEP) and the periphery of the AF, but these vessels do not extend further into the interior of the AF or the central NP, creating a hypoxic microenvironment for the NP [177]. Under chronic physiological hypoxia, macrophages exhibit more active autophagy, characterised by accelerated internalisation and degradation of ACs [178]. This regulatory mechanism of macrophages is crucial for the cell clearance process and further promotes the autophagic activity of macrophages in the degeneration of IVD against apoptotic NPCs. By deeply understanding the mechanism of macrophage autophagy under hypoxic conditions, we may reveal new biological pathways, offering not only new ideas for alleviating and treating IDD but also new perspectives and strategies for treating diseases related to hypoxia.

### 5.4 Regulation of Efferocytosis May Require Combination With Other Therapeutic Approaches

The regulation of efferocytosis may need to be combined with other therapeutic approaches, such as drugs, biomaterials, gene therapy, and so forth. Research has shown that dexmedetomidine can alleviate sepsis‐related acute lung injury by promoting macrophage efferocytosis [179]. Hydrogel‐coated phagocytosis inhibitors, by modulating the function of peritoneal macrophages, coordinate the polarisation, autophagy and phagocytosis of peritoneal macrophages, paving a new path for ovarian cancer treatment [180]. Effero‐RLP, a novel biomimetic liposome system that mimics the efferocytosis of macrophages, combines the membrane of apoptotic red blood cells (RBC) with liposomes to achieve efficient targeting of macrophages used to suppress inflammatory responses [181]. The chimeric receptor for efferocytosis designed by Sho Morioka can promote autophagy and inhibit inflammation [182]. Currently, a multitude of therapeutic strategies leveraging efferocytosis is in the experimental stage, and numerous drugs targeting autophagy for disease treatment are under continuous research [183]. Notably, statins are instrumental in modulating autophagy, particularly through pathways such as Rac‐1 and RhoA [184]. Integrated and simulated exocytosis‐functioning nanovesicles can promote M2 macrophage polarisation, regulate the imbalance between Treg and Th17 cells, and effectively inhibit the progression of rheumatoid arthritis (RA) [185]. Zhou et al. developed an IVD circular microneedle delivery system capable of transporting receptor‐M‐like engineered macrophages into the IVD, thereby enabling the clearance of apoptotic NPCs, reduction of inflammation and delay of degenerative progression through enhanced efferocytosis [186].

## 6 Conclusion

As pioneers of cellular immunity, macrophages serve a crucial function by engulfing and digesting pathogens, ACs and other foreign particles through a process called phagocytosis. They are essential for the body's immune defence and also secrete a variety of cytokines and chemical signals to regulate immune responses and promote tissue repair. In healthy discs, particularly within the NP, macrophage markers such as CD68 are minimally expressed due to the disc's avascular nature and immune‐privileged environment. However, during the progression of disc degeneration, macrophage infiltration markedly increases, particularly in regions of AF rupture or tissue injury. Macrophages, as pivotal immune effector cells, differentiate into distinct functional subtypes in response to environmental cues. In the context of IVD degeneration, these cells act both as ‘destroyers’ and ‘healers’, with their roles largely determined by their polarisation state and interactions with the local microenvironment. Under stimulation from LPS and IFN‐γ, macrophages adopt the M1 phenotype (pro‐inflammatory). M1 macrophages release pro‐inflammatory cytokines, including TNF‐α, IL‐1β and IL‐6, which amplify local inflammation, induce oxidative stress and promote ECM degradation, thereby accelerating disc degeneration. In contrast, exposure to molecules such as IL‐4 promotes macrophage polarisation into the M2 phenotype (anti‐inflammatory). M2 macrophages counteract pathological damage by suppressing inflammation, enhancing ECM synthesis and facilitating tissue remodelling, thereby contributing to the restoration of microenvironmental homeostasis within the disc.

In human IVD, the presence of macrophage markers CCR7 and CD163 is strongly associated with disc degeneration, indicating an increase in M1 phenotype (CCR7+) and M2c phenotype (CD163+) macrophages as IVD degeneration progresses [127]. However, current research has largely focused on disc degeneration and macrophage polarisation phenotypes, overlooking the fundamental process of macrophage phagocytosis of ACs. In degenerated IVD with a high number of ACs, efferocytosis plays a crucial role in IDD.

This process involves the engulfment and digestion of cells, aiding in the clearance of damaged ACs and modulating the inflammatory response of disc cells. Efferocytosis is a multi‐step process that begins with macrophages recognising ‘find‐me’ signals released by ACs, such as membrane lipids, nucleotides, and chemokines. This is followed by the detection of ‘eat‐me’ signals on the apoptotic cell surface, including PtdSer and specific protein molecules.

Once the ACs are identified, macrophages engulf and digest their contents. During this process, macrophages release anti‐inflammatory cytokines like IL‐10 and TGF‐β while suppressing the secretion of pro‐inflammatory cytokines such as TNF‐α and IL‐1β. This coordinated response helps to dampen inflammation and promote tissue repair. Additionally, efferocytosis shifts macrophages towards an anti‐inflammatory M2 polarisation state, inhibiting the pro‐inflammatory M1 polarisation. By reducing the release of inflammatory mediators, efferocytosis can lower the inflammatory state of disc cells, thereby slowing the progression of IDD. Therefore, based on the therapeutic applications of efferocytosis in other diseases, we hypothesise that it could also serve as a novel treatment approach for IDD, warranting further investigation.

In summary, efferocytosis offers a new perspective for IDD treatment by regulating the inflammatory response, promoting cell survival and proliferation and enhancing repair. Future research may uncover more about the mechanisms of efferocytosis in IDD, providing a scientific basis for developing new therapeutic strategies to delay or reverse disc degeneration.

Despite the current lack of a deep understanding of the specific mechanisms of macrophage efferocytosis in IDD, this has led to the absence of targeted therapeutic approaches for this function. However, it is precisely this underexplored area that reveals the great potential of macrophage efferocytosis as a potential therapeutic strategy for IDD. Through further research, we hope to unlock the key role of macrophages in maintaining the health of the IVD, paving new paths for the prevention and treatment of IDD. This in‐depth study of efferocytosis not only enriches our understanding of the pathological mechanisms of IDD but may also lead to innovative treatment methods, thereby improving the quality of life for patients.

While this manuscript explores efferocytosis as a potential therapeutic strategy for IDD, significant challenges remain. The most critical limitation is the lack of experimental evidence supporting the efficacy of efferocytosis in IDD treatment. Additionally, potential risks, such as excessive tissue remodelling, fibrosis or immune dysregulation, have not been thoroughly examined. Although various therapeutic approaches targeting efferocytosis are discussed, the manuscript lacks detailed strategies for their practical implementation, optimisation, and integration with existing IDD treatments. Therefore, further experimental research is essential to validate the therapeutic potential of efferocytosis and assess its clinical feasibility.

## Author Contributions

Shijie Chen, Haijun Zhang, Zhaoheng Wang, Daxue Zhu, and Yanhu li were co‐first authors who contributed equally to this article. Shijie Chen, Haijun Zhang, Yanhu li, and Daxue Zhu put on the reference collection, reference analysis and manuscript writing. Zhaoheng Wang, Yizhi Zhang and Dongxin Wang contributed to the topic conception. Xuewen Kang, the corresponding author, contributed to revising the manuscript and figures and decided to submit them for publication.

## Conflicts of Interest

The authors declare no conflicts of interest.

## Data Availability

Data sharing not applicable to this article as no datasets were generated or analysed during the current study.
